# Trial Files: Leveraging large language models to summarize practice-changing clinical trials for clinicians

**DOI:** 10.1371/journal.pone.0351135

**Published:** 2026-08-07

**Authors:** Katarina Zorcic, Emily Bartsch, Bryant Lim, Genevieve McCallum, Tamara Van Bakel, Alison Hacker, Michael Fralick

**Affiliations:** 1 Lunenfeld-Tanenbaum Research Institute, Sinai Health System, Toronto, Ontario, Canada; 2 School of Medicine, Queen’s University, Faculty of Health Sciences, Kingston, Ontario, Canada; 3 Division of Internal Medicine, Temerty Faculty of Medicine, University of Toronto, Toronto, Ontario, Canada; 4 Department of Anesthesiology, Pharmacology, and Therapeutics, Faculty of Medicine, University of British Columbia, Vancouver, British Columbia, Canada; Christian Medical College Vellore, INDIA

## Abstract

**Background:**

Each day, over 100 randomized controlled trials (RCTs) are published, making it impossible for clinicians to stay up-to-date with medical literature. Large language models (LLMs) can identify and summarize emerging clinical evidence and support medical education.

**Methods:**

We created and prospectively evaluated a newsletter, Trial Files, which leverages an LLM to summarize RCT abstracts relevant to general internal medicine. We created a software tool, called PaperScrape, which leverages the Medline application programming interface (API) to identify trials published in five high-impact journals. Information from each RCT’s abstract was extracted, and plain-language summaries were generated using OpenAI’s LLM API. We analyzed the accuracy of summaries generated by an LLM (compared to manual review), results of a subscriber survey, and effectiveness of marketing strategies on user growth.

**Results:**

From June 2023 to March 2025, 50 newsletters with 3 RCTs each were distributed to 648 subscribers. A subset of 96 RCTs was randomly selected to evaluate reporting accuracy with prompt engineering. The accuracy for reporting study information with prompt engineering, compared to manual review, was 97.1% for study phase, 92.2% for blinding, 85.4% for sample size, 97.9% for patient population, 94.7% for comparison groups, and 92.7% for primary outcome. Forty-three subscribers completed a survey about Trial Files. The mean overall rating was 4.7 out of 5 (5 representing “very good”), and all respondents agreed the newsletter made it easier to keep up-to-date with emerging clinical trials in internal medicine. The most effective strategy for user growth was promotion at a meeting, conference, or education session (6.8 subscribers per day, compared to 0.7 subscribers gained per day on days without promotion, p < 0.0001).

**Conclusion:**

LLMs can provide concise, accurate summaries of RCTs, which can help general internists stay up-to-date on recently published trials.

## Background

Evidence-based medicine is the practice of integrating clinical judgment with current best evidence, and is essential for making informed clinical decisions and improving patient care.[1–3] In 2010, 75 randomized controlled trials (RCTs) and 11 systematic reviews were published daily, with no anticipated plateau in the projected growth of publications.[4] Nearly 10 years later, the number of RCTs and systematic reviews published each day were estimated to be approximately 140 and 80, respectively. [5–7] This volume of publications far exceeds the available time of clinicians and medical trainees. For trainees in particular, it can be overwhelming to become familiar with landmark, practice-setting RCTs while concurrently assimilating emerging evidence into their clinical workflows.[2,4,8] According to a study of American Emergency Medicine resident physicians, keeping up with medical literature was reported to be one of their most significant day-to-day challenges.[8] While clinicians commonly access resources such as UpToDate or MedScape to find summaries of the existing literature, their content may exclude recently published trials and the cumbersome nature of their interfaces is not always user-friendly.[9–11] While systematic reviews can help keep clinicians up to date, they commonly lag behind the most recent RCTs by one year, are time-consuming to review, and are typically outdated by the time of publication.[2,12] Evidently, novel strategies to collect, summarize, and share clinical research are needed.

Artificial intelligence (AI) has the potential to modernize medical practice, including medical education, clinical decision-making, and healthcare research.[13–15] In medical education, specific applications of AI-based large language models, such as ChatGPT, include summarizing medical research, generating realistic patient simulations, personalizing learner experiences, and enhancing medical textbooks.[13,14] Additionally, the integration of AI tools into medical education programs can improve students’ digital and AI literacy skills.[16] A cross-sectional study of graduates from an international medical school showed that 63% of medical trainees planned to use AI during residency to explore new medical topics and research.[17] Accessible resources that inform clinicians of new research are needed. Our objective was to evaluate the accuracy and usefulness of a large language model to generate a newsletter summarizing results from RCTs.

## Methods

### Study design

This study represents a prospective implementation and evaluation of an LLM-assisted knowledge-translation system. We prospectively created and evaluated a newsletter summarizing RCTs relevant to general internal medicine using a large language model. We selected the following journals to identify RCTs: New England Journal of Medicine, Annals of Internal Medicine, Journal of the American Medical Association (JAMA), JAMA Internal Medicine, and The Lancet. These were selected because they commonly publish RCTs relevant to general internal medicine. This focused selection was intended to prioritize high-relevance trials for early implementation of the PaperScrape tool. The primary evaluation outcome was the accuracy of LLM-generated summaries compared with source abstracts. Secondary outcomes included subscriber growth and user-reported usefulness.

### Data pipeline

**Fig 1:**
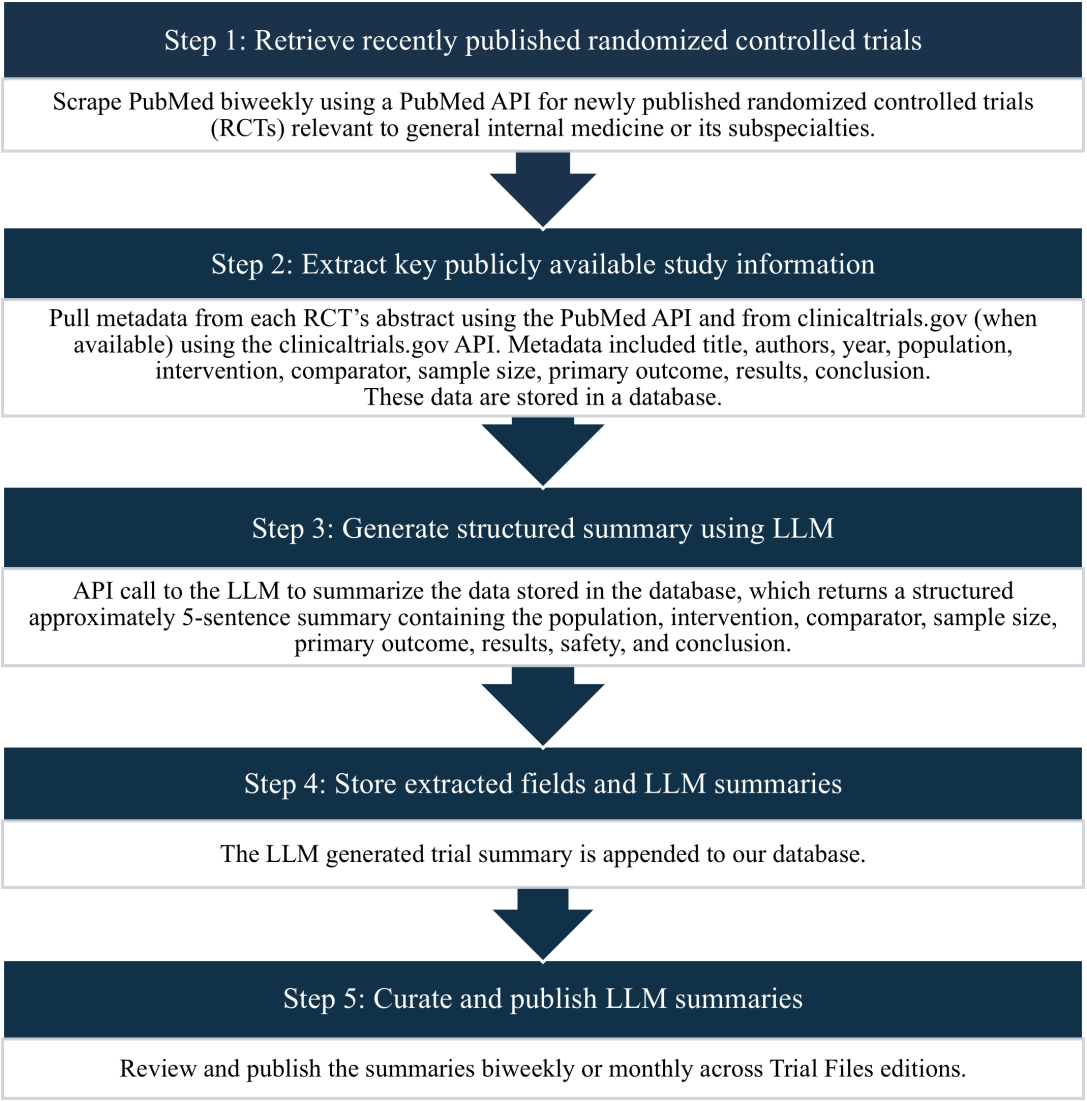
Trial Files data processing and LLM summarization pipeline.

To identify newly published RCTs from the aforementioned journals, we created a software tool called PaperScrape. PaperScrape leverages Medline’s E-utilities ESearch application programming interface (API) to identify published RCTs. Trials were identified using the following keywords: randomized controlled trial [publication type], clinical trial [publication type], and randomized trial [title]. After the trials were identified, we excluded those published in oncology, as they were deemed unlikely to be relevant to general internal medicine. We then scraped the data from the abstract for included trials, which included all data reported in the abstract, including the ClinicalTrials.gov number (when reported) (Fig 1). We did not extract data from the full manuscripts because they were typically behind a paywall.

### Leveraging a large language model

The scraped data was stored in a Google Sheet. The abstract text and a prompt served as inputs into OpenAI’s large language model API, gpt-3.5-turbo-instruct (released in late 2023; see model documentation: https://platform.openai.com/docs/models/gpt-3.5-turbo-instruct), which was used to generate all clinical trial summaries between June 2023 and March 2025. Open AI’s large language model API changed over time and included text-davinci-003 (June 2023 to October 2023), gpt-3.5-turbo-instruct (October 2023 to February 2025), and gpt-40-mini (February 2025 to date). All summaries were generated using consistent model settings. The system instruction provided to the model was, *‘You are a helpful physician assistant.’* We used a temperature of 0.4, a maximum token limit of 1400, and set *n* to 1. The nucleus sampling parameter *top_p* was set to 1, and both the frequency and presence penalties were set to 0. These hyperparameters remained constant for all trial summaries produced in the study. An illustrative example of an original RCT abstract and associated LLM-generated summary is shown in S1 File.

### Prompt engineering

Prompt engineering refers to the process of developing, iterating, and refining the instructions (“prompts”) you give to an LLM in order to increase the quality and accuracy of its outputs. Summaries were generated by ChatGPT initially using a baseline prompt and later using an engineered prompt. We compared the reporting accuracy of study information of the baseline and engineered prompts. The baseline prompt entered into ChatGPT was: “Summarize this abstract [abstract text]”. The engineered prompt included instructions to standardize the structure and information included in the summary. The engineered prompt incorporated explicit instructions specifying which trial elements to summarize and included criteria such as ‘do not output information for a given domain if it is not reported in the abstract,’ an iterative process we used to reduce hallucinations and standardize output structure. The full engineered prompt is included in S2 File. Prompt engineering was conducted iteratively during development, after which the final engineered prompt was fixed prior to the accuracy evaluation phase and applied consistently to all summaries analyzed.

### LLM summary accuracy evaluation

Trial characteristics were manually compared between each publication abstract and its corresponding LLM summary in a binary fashion by one human reviewer (BL). A trial characteristic was considered accurate if it was both present and correctly represented in the LLM summary. For example, trial phase was coded as inaccurate if the LLM summary reported a different phase than the one stated in the abstract. Accuracy for each domain was calculated as the proportion of characteristics correctly reported out of the total assessed.

### Hallucination analysis

To evaluate the accuracy of the current Trial Files LLM summaries, we conducted a structured hallucination analysis of 30 randomly selected RCT summaries generated between September and October 2025. Each summary was compared to its corresponding published abstract across seven predefined domains: sample size, patient population, intervention, comparator, primary outcome, blinding, and placebo.

One independent annotator (KZ) extracted each domain directly from the original abstract and compared it with the corresponding content in the LLM-generated summary. A hallucination was defined as any fabricated, incorrect, or unsupported statement. Each domain was scored using a standardized rubric as Y if the information in the summary differed from the abstract, N if it matched the abstract or correctly stated that the information was not reported, and NA if the summary lacked the domain when it was present in the abstract. Hallucination frequency was calculated as the proportion of summaries with at least one Y for a given domain.

### Dissemination of Trial Files

The first issue of the Trial Files newsletter was released on June 2, 2023, and subsequent issues were released every two weeks freely through Substack, a subscription-based publishing platform. Each issue of Trial Files included three RCTs, and issues alternated between sharing newly-published RCTs, which were selected from the PaperScrape output, and “throwback” RCTs, which were manually-selected RCTs published at least three years prior to the newsletter release and that the study authors deemed as practice-changing in the field of general internal medicine. All LLM-generated summaries were reviewed by a clinician prior to newsletter dissemination to ensure consistency with the source abstract.

Following the release of Trial Files, several subspecialty arms of Trial Files were created: Cardio Trial Files, Nephro Trial Files, Thrombo Trial Files, Inflammatory Bowel Disease (IBD) Trial Files, and Diabetes Trial Files. The data pipeline for each of these was the same as outlined above, except that core journals accessed by PaperScrape differed by specialty. Article selection for these subspecialty newsletters was done by dedicated subspecialist teams.

Trial Files was promoted using several marketing strategies, including word of mouth, email (predominantly to internal medicine training programs and organizations), posts on Twitter/X, educational sessions/conferences, and paid advertisements on Reddit.

### Subscriber growth

#### Analysis of overall subscriber growth.

Substack recorded the number of views Trial Files received daily. We calculated the mean number of views per month from June 2023 to March 2025. Substack further divided the number of views based on their source (email subscriptions, direct links, Substack app, and other). We calculated the proportion of views from each source from June 2023 to March 2025.

#### Analysis of subscriber growth according to promotional strategy.

The increase in subscriber number following a promotional strategy was evaluated. Subscriber gain was divided into 5 promotional categories (emails, tweets, education sessions, paid advertisements, and other), and the increase in growth of daily subscribers was calculated.

### Trial files feedback

In February 2025, an optional survey was sent by email to subscribers to elicit feedback about Trial Files. The survey link could also be accessed through the last Trial Files newsletter from that month and the following two Trial Files newsletters from March 2025. Coffee gift cards valued at $10 CAD were offered to the first 40 respondents. The survey questions can be found in S1 Table. The subscriber survey was anonymous and required participants to provide informed consent electronically before proceeding. This consent process and survey were approved by the Mount Sinai Hospital Research Ethics Board**.**

### Statistical analysis

Subscriber growth from June 2, 2023 to March 31, 2025 and projected subscriber growth, assuming continued current pattern of promotion, were modelled with an ARIMA(1,1,1) time series model. We selected the ARIMA(1,1,1) model using Akaike Information Criterion minimization, verified by the auto.arima function, and confirmed adequate model fit by inspecting ACF/PACF residual plots and ensuring the absence of residual autocorrelation.[18] The number of subscribers gained the day following a promotional strategy were compared using a one-sided Mann-Whitney-Wilcoxon test with a Bonferroni correction (alpha = 0.01). All statistical analyses were performed using R.

## Results

### Accuracy of summaries

Summaries were generated from 96 randomly selected RCT abstracts. The reporting accuracy of study information using the baseline prompt, compared with manual review of abstracts, was 54.5% for study phase, 59.7% for blinding, 44.8% for sample size, 91.7% for patient population, 75.8% for comparison groups, and 84.4% for primary outcome. Following prompt engineering, the accuracy of reporting increased to 97.1% for study phase, 92.2% for blinding, 85.4% for sample size, 97.9% for patient population, 94.7% for comparison groups, and 92.7% for primary outcome. Four summaries (4.2%) included information that was not present in the abstract.

### Hallucination frequency

Across 30 randomly selected RCT summaries generated between September and October 2025, hallucinations were rare across all evaluated domains. Hallucination frequencies were 3.3% for sample size, 0% for patient population, 0% for intervention, 6.7% for comparator, 3.3% for primary outcome, 0% for blinding, and 0% for placebo.

### User survey results

The survey was distributed between February 20 and March 31, 2025. There were 43 subscribers who responded (Table 1). The mean overall rating of the Trial Files newsletter was 4.7 out of 5 (SD = 0.46) (with a score of 1 representing “very poor” and a score of 5 representing “very good”). When asked whether Trial Files newsletters make it easier to keep up to date with emerging clinical trials in internal medicine, 60% (n = 26 of 43) strongly agreed, 37% (n = 16 of 43) agreed, and 2% (n = 1 of 43) expressed neutrality. When asked whether the throwback issues of Trial Files newsletters help to understand the evidence base for current practices in internal medicine, 49% (n = 21 of 43) strongly agreed, 44% (n = 19 of 43) agreed, and 7% (n = 3 of 43) expressed neutrality. When asked if the summaries were comprehensive and included the key information one would look for, 42% (n = 18 of 43) strongly agreed, 56% (n = 24 of 43) agreed, and 2% (n = 1 of 43) expressed neutrality. In terms of preference for the frequency of the newsletter, 56% (n = 24 of 43) of respondents preferred once weekly, 28% (n = 12 of 43) preferred every two weeks, 9% (n = 4 of 43) preferred twice weekly, and 7% (n = 3 of 43) preferred once monthly. When asked if they would like to see visual infographics alongside the current textual summaries, 88% (n = 38 of 43) of respondents said yes and 12% (n = 5 of 43) said no.

**Table 1:** Questions, response options, and responses (N = 43) of the survey sent to Trial Files subscribers in February 2025.

Questions	Response Options	Responses (%)	Responses (N)
What is your overall rating of the Trial Files Newsletter (1=very poor, 5=very good)?	Numerical rating of 1	0	0
	Numerical rating of 2	0	0
	Numerical rating of 3	0	0
	Numerical rating of 4	30	13
	Numerical rating of 5	70	30
The Trial Files newsletters make it easier for me to keep up to date with emerging clinical trials in Internal Medicine.	Strongly agree	60	26
	Agree	37	16
	Neutral	2	1
	Disagree	0	0
	Strongly disagree	0	0
The Throwback issues of Trial Files newsletters help me to understand the evidence base for current practices in Internal Medicine.	Strongly agree	49	21
	Agree	44	19
	Neutral	7	3
	Disagree	0	0
	Strongly disagree	0	0
The article summaries are comprehensive and include the key information I am looking for.	Strongly agree	42	18
	Agree	56	24
	Neutral	2	1
	Disagree	0	0
	Strongly disagree	0	0
Ideally, how frequently would you like to receive Trials Files updates?	Twice weekly	9	4
	Once weekly	56	24
	Every 2 weeks	28	12
	Monthly	7	3
Ideally, how many articles would be included in each issue?	Up to 3	56	24
	Up to 5	42	18
	Up to 10	2	1
Would you like to see visual infographic summaries alongside the current 3-5 sentence article summaries?	Yes	88	38
	No	12	5
Ideally, how many infographics would you like to see per newsletter?	None	0	0
	One	29	11
	Two	32	12
	Three	39	15

### Subscriber growth

**Fig 2:**
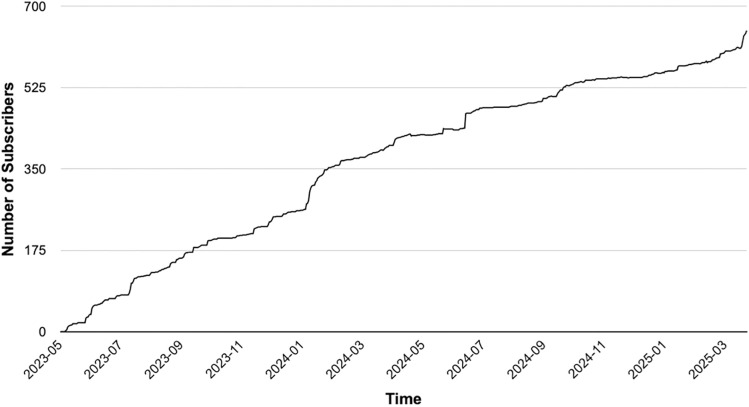
Trial Files subscriber growth (zero to 648 subscribers) between June 2, 2023 and March 31, 2025.

Between June 2, 2023 and March 31, 2025, subscribers increased from zero to 648 (Fig 2). The most common source of viewing was from email subscriptions (69%), followed by direct links (28%) and the Substack app (3%). Baseline growth without promotion was approximately 0.7 subscribers per day. Promotional strategies were associated with an increase in subscribers on the day the promotional strategy was employed, with the highest subscription rate following promotion at a meeting, conference, or education session (6.8 subscribers per day, p < 0.0001), followed by emails (2.4 subscribers per day, p < 0.001) and Twitter/X posts (1.7 subscribers per day, p = 0.0343).

Across subspecialty Trial Files during this time, there were an additional 622 subscribers to the subspecialty Trial Files: 198 subscribers to Thrombo Trial Files, 137 subscribers to Nephro Trial Files, 138 subscribers to Cardio Trial Files, 84 subscribers to IBD Trial Files, and 65 subscribers to Diabetes Trial Files. Mean number of views per month was 1417 for Trial Files, and views were highest on the day the newsletter was distributed. Mean monthly views were 218 for Cardio Trial Files, 308 for Nephro Trial Files, 322 for Thrombo Trial Files, 75 for IBD Trial Files, and 74 for Diabetes Trial Files.

## Discussion

We created a software tool called PaperScrape to identify RCTs from five high-impact journals, leveraged ChatGPT to write plain-language summaries from the abstracts, and disseminated the summaries in Trial Files, a free, twice-monthly newsletter targeted toward general internal medicine trainees and physicians.

AI has become an important facet of medical education, with applications already recognized in simulated patient encounters, scientific writing, exam preparation, and summarizing current evidence by performing literature reviews.[14,17,19] Our study reveals two emerging applications of AI in medical education. First, the development and implementation of PaperScrape simplified the process of identifying recently published RCTs. Second, we were able to generate highly accurate abstract summaries using a large language model. This finding was congruent with the results of previous studies, which showed that prompt engineering improved the consistency and reliability of outputs, thereby enhancing the overall performance of large language models.[20–22]

We observed an increase to 648 subscribers to Trial Files following its initial release, and interventions to promote the newsletter were found to be effective. Furthermore, interest in similar tools from subspecialty colleagues led to the development of several subspecialty arms of Trial Files. In response to the growth of our newsletter, we recognize the need for thoughtful integration of resources like Trial Files in medical education. The educational value of such AI-generated resources as our newsletter must be weighed against the potential limitations of AI, including bias, generation of false information, potential undermining of critical thinking skills, and other ethical considerations.[13,17,19,23] In addition, the uptake of AI-informed tools will need to correspond to curricular changes in medical training to ensure that formal guidance is provided on the appropriate uses, intrinsic deficiencies, and pitfalls of AI across different clinical applications.[17,23]

There are several strengths to our study. First, we provide a timely use case for how large language models can aid clinicians and medical trainees in staying up-to-date with the literature. Second, as evidenced by the creation of Trial Files for other specialties, this initiative is easy to spread and scale beyond general internal medicine. Third, the trial summaries were highly accurate following prompt engineering. Finally, we prospectively evaluated our tool by surveying subscribers, who reported that the newsletter was a valuable resource for staying up-to-date with clinical evidence.

Our study also has limitations. First, large language models can confabulate (sometimes referred to as “hallucinate”), and while the overall prevalence varies, it is approximately 3%.[14,17,24] This was consistent with what we observed in our study. Second, there is debate as to whether outsourcing the task of finding and summarizing RCTs may adversely affect the learning of medical trainees and degrade critical thinking. While we believe this risk is overstated, the learning needs of busy clinicians must be balanced against their other competing demands.[14,17,19] Third, our user survey only elicited 43 responses (about 7% of subscribers at that time), so there is a risk of response bias, as respondents may differ from non-respondents in engagement, satisfaction, or perceived usefulness. Additionally, because neither our survey nor our subscriber database collects demographic information (e.g., level of training), we were unable to compare respondents with the broader subscriber base to assess representativeness. Future studies should consider collecting demographic information to better evaluate generalizability. Fourth, we extracted information only from publicly accessible sources such as abstracts and ClinicalTrials.gov, as full-text manuscripts are frequently behind paywalls. By generating summaries limited to information from publicly available sources, the newsletters may not fully capture the nuances of the published RCTs. Fifth, the general internal medicine edition of Trial Files had an initial scope of five high-impact journals, which may limit representation of the broader clinical literature across specialties and journal tiers. Subspecialty Trial Files editions already incorporate additional specialty journals, and future iterations of the general edition may expand journal coverage to improve representativeness. Sixth, the evaluation of Trial Files focused on summary accuracy, subscriber growth, and perceived usefulness and did not assess downstream effects on clinician decision-making, practice behavior, or patient outcomes. Future studies should examine the clinical impact of LLM-assisted evidence dissemination.

An emerging consideration for future work is the application of reporting frameworks developed specifically for LLM-based tools. The recently proposed TRIPOD-LLM guidelines extend the traditional TRIPOD reporting principles to LLM applications, emphasizing transparency in data sources, model provenance, prompt design, evaluation datasets, and performance metrics.[25] Although Trial Files functions as a knowledge translation tool rather than a clinical decision support tool, many of these principles align with our methodological approach. In this study, we aimed to support transparency by detailing our data sources, summarization pipeline, prompt engineering methods, and accuracy evaluation protocol, and by providing access to the abstracts and extracted trial data through the T-CAIREM Health Data Nexus repository.

In conclusion, the rapid pace of publication of RCTs limits the ability of clinicians to remain current with medical literature. Large language models can generate accurate and concise summaries of RCTs. Future work is needed to understand how large language models can be leveraged to contribute to medical education and how curricula will need to be adapted to incorporate training in AI.

## Supporting information

S1 File Sample RCT Abstract and LLM Summary Used in the Trial Files Pipeline. (DOCX)

S2 File PaperScrape Engineered Prompt. (DOCX)

S1 Table Questions and response options sent to Trial Files subscribers in February 2025. (DOCX)
