# Supplementary material for: Trial Files: Leveraging large language models to summarize practice-changing clinical trials for clinicians
Source: PLoS One. 2026 Aug 7;21(8):e0351135. doi: 10.1371/journal.pone.0351135 (PMC13450780; doi:10.1371/journal.pone.0351135)
Supplement: Supplementary file 1 [file pone.0351135.s001.docx]

**S1 File. Sample RCT Abstract and LLM Summary Used in the Trial Files Pipeline.**

Title: Early Diagnosis and Treatment of COPD and Asthma – A Randomized, Controlled Trial

DOI: 10.1056/NEJMoa2401389

**Abstract:**

Background
Many persons with chronic obstructive pulmonary disease (COPD) or asthma have not received a diagnosis, so their respiratory symptoms remain largely untreated.

Methods
We used a case-finding method to identify adults in the community with respiratory symptoms without diagnosed lung disease. Participants who were found to have undiagnosed COPD or asthma on spirometry were enrolled in a multicenter, randomized, controlled trial to determine whether early diagnosis and treatment reduces health care utilization for respiratory illness and improves health outcomes. Participants were assigned to receive the intervention (evaluation by a pulmonologist and an asthma–COPD educator who were instructed to initiate guideline-based care) or usual care by their primary care practitioner. The primary outcome was the annualized rate of participant-initiated health care utilization for respiratory illness. Secondary outcomes included changes from baseline to 1 year in disease-specific quality of life, as assessed with the St. George Respiratory Questionnaire (SGRQ; scores range from 0 to 100, with lower scores indicating better health status); symptom burden, as assessed with the COPD Assessment Test (CAT; scores range from 0 to 40, with lower scores indicating better health status); and forced expiratory volume in 1 second (FEV1).

Results
Of 38,353 persons interviewed, 595 were found to have undiagnosed COPD or asthma and 508 underwent randomization: 253 were assigned to the intervention group and 255 to the usual-care group. The annualized rate of a primary-outcome event was lower in the intervention group than in the usual-care group (0.53 vs. 1.12 events per person-year; incidence rate ratio, 0.48; 95% confidence interval [CI], 0.36 to 0.63; P<0.001). At 12 months, the SGRQ score was lower than the baseline score by 10.2 points in the intervention group and by 6.8 points in the usual-care group (difference, −3.5 points; 95% CI, −6.0 to −0.9), and the CAT score was lower than the baseline score by 3.8 points and 2.6 points, respectively (difference, −1.3 points; 95% CI, −2.4 to −0.1). The FEV1 increased by 119 ml in the intervention group and by 22 ml in the usual-care group (difference, 94 ml; 95% CI, 50 to 138). The incidence of adverse events was similar in the trial groups.

Conclusions
In this trial in which a strategy was used to identify adults in the community with undiagnosed asthma or COPD, those who received pulmonologist-directed treatment had less subsequent health care utilization for respiratory illness than those who received usual care. (Funded by Canadian Institutes of Health Research; UCAP ClinicalTrials.gov number, NCT03148210.)

**LLM Summary:**

This multicenter, randomized, controlled trial aimed to determine if early diagnosis and treatment of undiagnosed COPD or asthma in adults reduces health care utilization for respiratory illness and improves health outcomes. A case-finding method was used to identify participants in the community, who were then randomized to receive either the intervention (evaluation by a pulmonologist and an asthma–COPD educator following guideline-based care) or usual care by their primary care practitioner. The primary outcome was the annualized rate of participant-initiated health care utilization for respiratory illness. Secondary outcomes included changes in disease-specific quality of life, symptom burden, and forced expiratory volume in 1 second. Of the 38,353 persons interviewed, 595 were found to have undiagnosed COPD or asthma and 508 were randomized. The intervention group had a significantly lower rate of primary outcome events compared to the control group. Additionally, at 12 months, the intervention group had improved quality of life and symptom burden, as well as increased forced expiratory volume. The incidence of adverse events was similar in both groups. In conclusion, early diagnosis and treatment with pulmonologist-directed care resulted in reduced health care utilization for respiratory illness in adults with undiagnosed COPD or asthma.
