# Supplementary material for: Trial Files: Leveraging large language models to summarize practice-changing clinical trials for clinicians
Source: PLoS One. 2026 Aug 7;21(8):e0351135. doi: 10.1371/journal.pone.0351135 (PMC13450780; doi:10.1371/journal.pone.0351135)
Supplement: Supplementary file 2 [file pone.0351135.s002.docx]

**S2 File. PaperScrape Engineered Prompt.**

The engineered prompt was as follows:

prompt=f'''You are a helpful physician assistant. Do these tasks for the following clinical trial abstract

- Sample size: return sum in digits only and remove "patients" or "participants".

- Intervention group: return this in a phrase or word and include dosages if applicable.

- Comparator group: return this in a phase or word.

- Patient population: do not include sample size.

- Primary outcome: short phrase with timepoint of evaluation if applicable.

- Primary outcome result intervention: return the results just for the intervention group, preferably in a number. Do not report statistical comparison with the control group.

- Primary outcome result comparator: return the results just for the intervention group, preferably in a number. Do not report statistical comparison with the control group.

- Primary outcome result overall: return statistical comparison between intervention and control groups.

- Safety outcome results

- Conclusion: one sentence summary.

- LLM Summary: return a four to five sentence structured summary of up to 140 words containing study type, study phase (if available), study duration, blinding (if available), patient population, comparison and intervention groups from the methods section, sample size of each group, primary outcomes with brief statistics, safety outcomes, and conclusion. Remove any information about funding source. Only include details that are present in the abstract, otherwise, omit the output.

{text}'''
