# Supplementary material for: Trial Files: Leveraging large language models to summarize practice-changing clinical trials for clinicians
Source: PLoS One. 2026 Aug 7;21(8):e0351135. doi: 10.1371/journal.pone.0351135 (PMC13450780; doi:10.1371/journal.pone.0351135)
Supplement: Supplementary file 3 [file pone.0351135.s003.docx]

**S1 Table. Questions and response options sent to Trial Files subscribers in February 2025.**

| **Questions** | **Response Options** |
| --- | --- |
| What is your overall rating of the Trial Files Newsletter (1=very poor, 5=very good)? | Numerical rating of 1, 2, 3, 4, or 5. |
| The Trial Files newsletters make it easier for me to keep up to date with emerging clinical trials in Internal Medicine. | Choice of strongly agree, agree, neutral, disagree, or strongly disagree. |
| The Throwback issues of Trial Files newsletters help me to understand the evidence base for current practices in Internal Medicine. | Choice of strongly agree, agree, neutral, disagree, or strongly disagree. |
| The article summaries are comprehensive and include the key information I am looking for. | Choice of strongly agree, agree, neutral, disagree, or strongly disagree. |
| Ideally, how frequently would you like to receive Trials Files updates? | Choice of twice weekly, once weekly, every 2 weeks, or monthly. |
| Ideally, how many articles would be included in each issue? | Choice of up to 3, up to 5, or up to 10. |
| Would you like to see visual infographic summaries alongside the current 3-5 sentence article summaries? | Choice of yes or no. |
| Ideally, how many infographics would you like to see per newsletter? | Choice of none, one, two, or three. |
| What improvements or changes would you like to see to Trial Files? All feedback and suggestions are welcome. | Free text. |
